# Dataset on rheological measurements of xanthan gum aqueous dispersions containing sodium chloride and settling dynamics of spheres and disks in these dispersions

**DOI:** 10.1016/j.dib.2022.108865

**Published:** 2022-12-28

**Authors:** Magdalena M. Mrokowska, Anna Krztoń-Maziopa

**Affiliations:** a Institute of Geophysics, Polish Academy of Sciences, Ks. Janusza 64, Warsaw 01-452, Poland; b Faculty of Chemistry, Warsaw University of Technology, Noakowskiego St. 3, Warsaw 00-664, Poland

**Keywords:** Rheology, Particle settling, Xanthan gum, Sedimentation, Non-Newtonian fluid, Salinity, Exopolymer

## Abstract

This paper presents a dataset collected in laboratory experiments on the settling of solid spheres and disks in shear-thinning and viscoelastic aqueous solutions of xanthan gum with sodium chloride addition. Two types of spheres with density of 1.41 g/cm^3^ varying in diameter (3.00 mm and 1.59 mm) and four types of disks with density of 1.43 g/cm^3^ and thickness of 0.3 mm varying in diameter (1.5 mm, 2.0 mm, 2.5 mm, and 3.0 mm) were considered. A single particle was settling in a column filled with a test solution which varied in salt content (from 0 M to 0.9 M), while xanthan gum content was constant (1 g/L). The total of elven solutions were tested. For each experimental set, a sequence of images with a falling particle was captured using a camera with macro lenses. Dataset includes position of particle in time and enables the evaluation of settling velocity. Rheological measurements were carried out for each test solution to assess flow properties and viscoelasticity. The following measurements were performed: shear dependent viscosity, shear stress amplitude sweeps, frequency sweeps, the dependence of the first normal stresses difference on shear strain at constant frequency (1 Hz). Datasets may be useful in various areas on fluid mechanics and rheology, e.g., in research on the impact of salinity on rheological properties of exopolymer solutions, to develop numerical models on solid particles settling in non-Newtonian fluids, and in studies on the impact of exopolymers and electrolytes dissolved in water on settling dynamics of solid particles.

**Specifications Table**

**Table:** 

Subject	chemistry
Specific subject area	sedimentation, rheology
Type of data	csv data
How the data were acquired	Rheological measurements were performed using Physica MCR 301 rheometer with parallel plate measuring geometry with a TrueGap® option (plate diameter 50 mm, 1 mm gap) and built-in Peltier device enabling precise temperature control. Images of particles settling in a transparent column were captured using Basler acA2500–60um camera with Schneider-Kreuznach macro lenses Componon 2.8/28–001 with aperture 3.5 F; 6 mm extension tube; images were recorded on a hard disk using digital video camera recording software StreamPix®. Images were pre-processed and processed using ImageJ and Matlab® Image Processing Toolbox to obtain data on temporal variation of particle centroid position.
Data format	Raw and filtered
Description of data collection	Rheological properties of solutions: - Flow curves; - Oscillatory tests at frequency 1 Hz under shear stresses 0.01 - 20 Pa; - Frequency sweeps in the range 0.01–10 Hz at constant shear stress amplitude 0.05 Pa; - The first normal stress difference dependence on shear strain at oscillation frequency 1 Hz. Settling experiments: Individual particles were released in the column. Images acquired using a camera were processed to identify the position of particle centroid.
Data source location	Institution: Laboratory of Hydrodynamic Micromodels, Institute of Geophysics, Polish Academy of Sciences City: Warsaw Country: Poland Institution: Warsaw University of Technology City: Warsaw Country: Poland
Data accessibility	Repository name: Mendeley Data Data identification number: 10.17632/ntgfb38k69.3 Direct URL to data [1]: 10.17632/ntgfb38k69.3
Related research article	M.M. Mrokowska, A., Krztoń-Maziopa, M. Dębowski. Effect of exopolymer gels on the viscoelasticity of mucus-rich saltwater and settling dynamics of particles, Marine Chemistry, 246 (2022) 104,163, 10.1016/j.marchem.2022.104163

## Value of the Data

• Dataset is useful in understanding the impact of salinity on the rheological properties of aqueous xanthan gum solutions and other exopolymer based systems as well as the effect of rheology on settling dynamics of spherical and non-spherical solid particles.
• Researchers studying rheological properties of fluids, especially solutions of polysaccharides, and settling processes in non-Newtonian fluids, representing the fields of rheology, food technology, petroleum, wastewater treatment, environmental sciences, oceanology can benefit from these data.
• These data may be used to develop and validate numerical models on the settling of spheres and disks in shear-thinning and viscoelastic solutions. Moreover, they can be used to analyse the impact of salinity on rheology of ionic aqueous exopolymer solutions.

## 1 Objective

These data were collected as a part of experimental research on the impact of rheological properties of saltwater with exopolymers on settling dynamics of particles to model marine processes in mucus-rich conditions [2]. This topic relates to the biologically modified properties of seawater with algal exopolymers that gain increasing interest in environmental studies [3,4].

However, the dataset presented herein in detail, has potential to be used in various rheology-related studies. Functional properties of exopolymers have been examined with reference to a wide range of applications in food processing, cosmetics, petroleum technology, mining and mineral processing, wastewater treatment, to name a few [5], [6], [7].

## 2 Data Description

Data presented in this paper refer to eleven experiments on particle settling in ionic aqueous solutions of xanthan gum. Datasets comprise the position of settling particle centroid in time and rheological properties of test solutions.

### 2.1 Settling Experiments

**Fig. 1:**
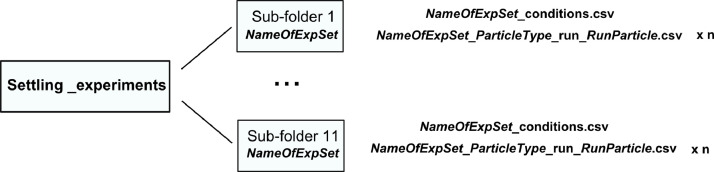
Data structure for settling experiments (explained in text), n indicates the total number of runs in each experimental set.

In settling experiments, the following data were produced: (1) 2D coordinates (horizontal and vertical) of particle centroid position varying in time, (2) data on experimental conditions including frame rate of image recording, solution temperature, type of particle. The data from settling experiments are collected in **Settling_experiments** folder and are organized according to the scheme shown in Fig. 1.

Eleven sets of settling experiments are provided, in sub-folders ***NameOfExpSet***, varying in the composition of test solution. In the names of folders and files, experimental sets are referred to as *XG+NaCl_x; NaCl_x+XG* indicating the order of addition of sodium chloride (NaCl) and xanthan gum (XG) to the solution and *x* indicates molar concentration of NaCl in a solution - a number within the range (0, 0.9). See Section 2.1.1. and Table 1 for details.

**Table 1:** Number of settling tests for each type of particle.

NameOfExpSet	ParticleType					
	S_3	S_1.5	D_3	D_2.5	D_2	D_1.5
NaCl_0+XG	15	10	11	11	11	10
NaCl_0.1+XG	6	6	6	6	6	4
NaCl_0.3+XG	7	5	5	4	5	5
NaCl_0.4+XG	6	4	5	4	5	5
NaCl_0.5+XG	9	6	6	5	5	4
NaCl_0.7+XG	6	5	4	4	5	3
XG+NaCl_0.1	6	5	5	6	5	3
XG+NaCl_0.3	6	5	5	5	5	3
XG+NaCl_0.5	5	5	5	4	5	3
XG+NaCl_0.7	8	6	5	4	6	2
XG+NaCl_0.9	5	5	5	5	5	2

Each sub-folder contains ***NameOfExpSet*_conditions.csv** file with 4 columns: *run, run_particle, frame_rate [fps], particle_type*, where *run* column includes the number of experimental run, *run_particle* indicates the number of run for a particular type of particle, *particle_type* includes information on the particle type in the following format: *P_d* where *P* indicates type of particle (*S* – sphere, *D* – disk) and *d* indicates the particle diameter in millimetres.

Moreover, each sub-folder contains several .csv files (equal to the number of all runs in the experimental set) called ***NameOfExpSet*_*ParticleType*_run_*RunParticle*.csv**, where *ParticleType* is in the following format *Pd* where *P* indicates type of particle (*S* – sphere, *D* – disk) and *d* indicates the particle diameter in millimetres, R*unParticle* indicates the number of run for the particular type of particle. Files contain data on the position of particle centroid in time in three columns: *x [pix], z [pix], t[s]*, where *x, z* are horizontal and vertical coordinates, respectively, in pixels and *t* is a time instant in seconds.

### 2.2 Rheology

**Fig. 2:**
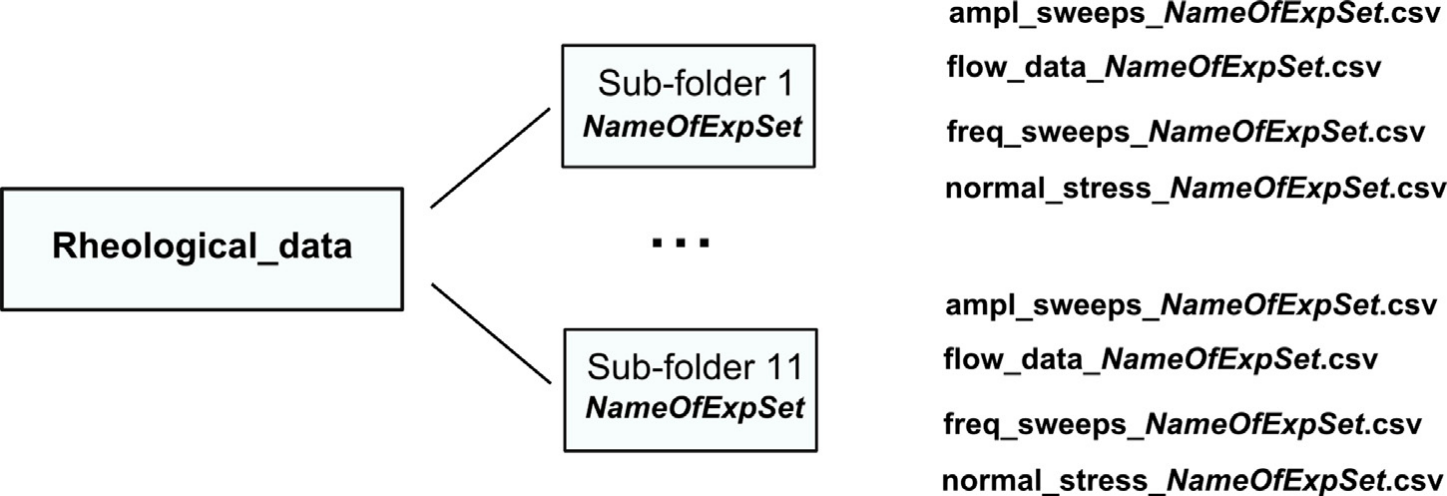
Data structure for rheology measurements.

In rheological tests, flow properties and viscoelastic properties of test solutions were measured. Data are collected in **Rheology_data** folder and are organized according to the scheme shown in Fig. 2. Data for eleven experimental sets are provided in sub-folders **Sub-folder 1 … Sub-folder 11** as shown in the scheme in Fig. 2. Naming convention for sub-folders is the same as explained in Section 1.1.

Each sub-folder contains four .csv files. Flow properties are collected in **flow_data.csv** containing four columns: *shear rate [1/s], shear stress [Pa], viscosity [Pa s], torque [μN m]*. Data from amplitude sweeps conducted at frequency 1 Hz under shear stresses varying from 0.01 to 20 Pa are collected in **ampl_sweeps.csv** file containing four columns: *strain [%], shear stress [Pa], storage modulus [Pa], loss modulus [Pa]*.

Data from frequency sweeps conducted in the range 0.01–10 Hz at constant shear stress amplitude (0.05 Pa) are collected in **frequency_sweep.csv** file containing three columns: *frequency [Hz], storage modulus [Pa], loss modulus [Pa].* Measurements of the first normal stress difference are collected in **normal_stress.csv** file containing three columns: *strain [%], 1st Norm. Str. Diff. (Lodge-Meissner) [Pa], shear stress [Pa].*

## 3 Experimental Design, Materials and Methods

In the experiments, data on settling of solid spheres and disks in ionic aqueous solutions of xanthan gum were collected and rheological properties of test solutions were measured. In the following Sections 2.1 and 2.2, materials, settling experiments conditions and set-up, and rheological tests are described.

### 3.1 Materials

#### 3.1.1 Solutions

Test solutions were prepared in Laboratory of Hydrodynamic Micromodels, Institute of Geophysics, Polish Academy of Sciences. The solutions comprised of distilled water, xanthan gum commercial food powder and sodium chloride (pure for analysis). The concentration of xanthan gum was constant (1 g/L) while the concentration of NaCl varied from 0 M and 0.9 M. Two sets of samples ‘XG+NaCl’ and ‘NaCl+XG’ were prepared varying in the sequence of constituents addition (which resulted in different rheological properties).

Two litres of each solution was prepared as follows. In the case of ‘XG+NaCl’ samples, XG powder was mixed to the half of volume of distilled water, i.e., 1 L. The other half volume of water was used to prepare ionic solutions by adding NaCl in amounts to get appropriate molar concentrations. Then, two solutions were mixed and distilled water was added if necessary to get the final volume of 2 L. In ‘NaCl+XG’ solutions, NaCl aqueous solution was prepared in the final volume of distilled water, i.e., 2 L and then aliquots of XG powder were added to the solution and left to hydrate for 3 days and next mixed.

#### 3.1.2 Particles

Spheres and disks were considered in the experiments. Two types of commercially available spheres made of POM (polyoxymethylene) with density of 1.41 g/cm^3^ with varying diameter (3.00 mm and 1.59 mm) and four types of disks made of PCV (polyvinyl chloride) with density of 1.43 g/cm^3^, thickness of 0.3 mm varying in diameter (1.5 mm, 2.0 mm, 2.5 mm, and 3.0 mm) were used. Disks were manufactured from PCV foil. In each experiment, a few particles of the same type were tested.

### 3.2 Settling Experiments

**Fig. 3:**
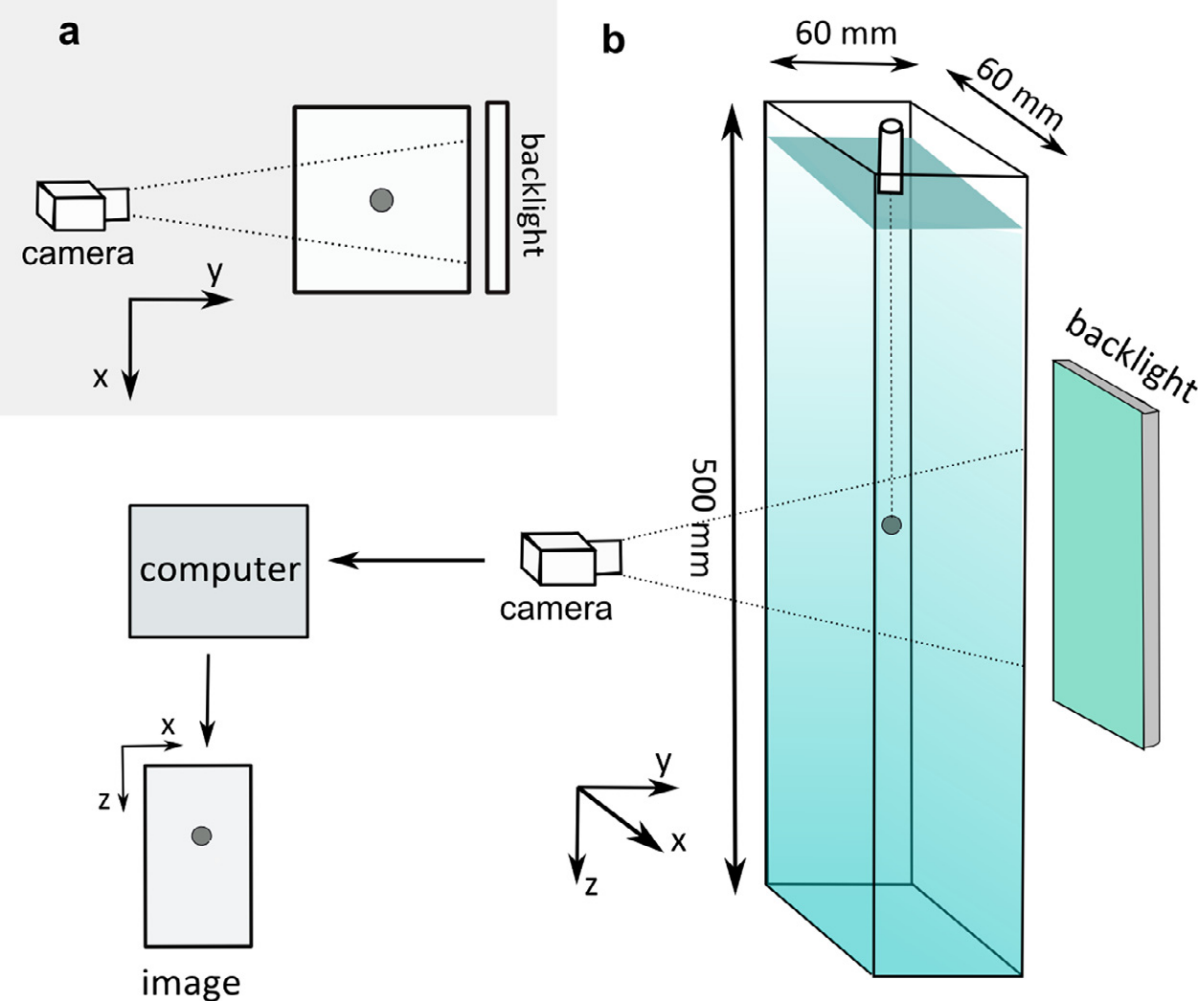
Scheme of settling experiment set-up. (a) horizontal view, (b) 3D view of set-up (not to scale).

Settling experiments were performed in the Laboratory of Hydrodynamic Micromodels, Institute of Geophysics, Polish Academy of Sciences. Experiments were carried out in a transparent polycarbonate column with inner cross section 0.060 m x 0.060 m and a height of 0.5 m (see Fig. 3). A column was gently filled with a test solution before each experiment to avoid trapping of air bubbles. The column was backlit using the LED panel for uniform illumination. During an experiment, temperature of solution was controlled a few times using a liquid thermometer to an accuracy of 0.1 ⁰C.

A particle was introduced beneath the surface of liquid in the centre of column cross section using a twizzer. To ensure precise release of particles, spheres were released into a 4 mm-diameter tube partially immersed in a test solution. To introduce disks to the settling column, a vertical plate was placed vertically and partially immersed in a test solution to release a disk in a vertical position so that the disk touched its surface and slid until it has been released into the test solution. A particle was settling freely in a solution under gravity.

A single experiment was dedicated to test settling of all types of particles in a particular type of solution. Each experiment comprised several runs. During each run, a single particle was released and recorded. Particles were released in a sequence and a few particles of the same type were released as shown in Table 1 (e.g., for experiment NaCl_0+XG: 15 POM_3 particles, next 10 POM_1.5 particles, next 11 PCV_3 particles were released). Particles were released every few minutes to ensure that a solution had relaxed.

A particle settling in the column was recorded using monochromatic Basler camera acA2500–60um with Schneider-Kreuznach macro lenses Componon 2.8/28–001 with aperture 3.5 F and 6 mm extension tube operating at 2590 x 2048 resolution. Micrometre reading up to 0.01 mm was used to assess spatial resolution of images which was 31 μm per pixel.

The camera was fixed in front of the column perpendicular to the wall in a distance of 160 mm from the front wall. The centre of field of view was positioned about 350 mm below the position of the surface of test solution, deep enough to ensure terminal conditions for particle settling and the lack of residual effects of particle release.

Monochromatic images were acquired at frequency adjusted for each type of particle in a test recording. Camera parameters including acquisition frame rate and exposure time were controlled via digital video camera recording software StreamPix® and the images were recorded directly on the computer hard disk. To reduce data volume, images were next cropped to remove an area outside the particle path, the width of an image was reduced while vertical dimension was preserved. Rolling ball background subtraction available in ImageJ using ‘radius = 200′ was applied to clear out the background noise of each image. In a following step, digital thresholding available in Matlab® was applied to convert images into binary mode and coordinates of particle centroid were identified in each image using Image Processing Toolbox.

### 3.3 Rheological Experiments

Rheological experiments were performed at Department of Inorganic Chemistry of Warsaw University of Technology, using rotational rheometer Physica MCR301. Measurements were performed in a parallel plate geometry with a TrueGap system, at temperature 21 °C. Before each measurement every sample was equilibrated 5 min in the measuring system. To avoid evaporation of the solvent during measurements the dedicated solvent trap system was used. Flow curves were recorded in controlled shear rate mode in the shear rate ranging from 0.01 to 1000 *s* ^−^ ^1^. Small strain oscillatory tests were performed at constant and variable frequencies to derive the linear viscoelastic range and relaxation times of the investigated materials. Experimental data were analysed with the calculation modules embedded in a Rheoplus software.

## Ethics Statements

Not applicable.

## CRediT authorship contribution statement

**Magdalena M. Mrokowska:** Conceptualization, Methodology, Data curation, Visualization, Writing – original draft, Writing – review & editing. **Anna Krztoń-Maziopa:** Methodology, Data curation, Writing – original draft, Writing – review & editing.

## Declaration of Competing Interest

The authors declare that they have no known competing financial interests or personal relationships that could have appeared to influence the work reported in this paper.

## Data Availability

Dataset on rheological measurements of xanthan gum aqueous dispersions containing sodium chloride and settling dynamics of spheres and disks in these dispersions (Original data) (Mendeley Data)
